# Molecular basis of human angiotensin‐1 converting enzyme inhibition by a series of diprolyl‐derived compounds

**DOI:** 10.1111/febs.17384

**Published:** 2025-01-06

**Authors:** Kyle S. Gregory, Gyles E. Cozier, Stephen Fienberg, Kelly Chibale, Edward D. Sturrock, K. Ravi Acharya

**Affiliations:** ^1^ Department of Life Sciences University of Bath UK; ^2^ Holistic Drug Discovery and Development (H3D) Centre University of Cape Town Rondebosch South Africa; ^3^ South African Medical Research Council Drug Discovery and Development Research Unit, Department of Chemistry and Institute of Infectious Disease and Molecular Medicine University of Cape Town Observatory South Africa; ^4^ Department of Integrative Biomedical Sciences, and Institute of Infectious Disease and Molecular Medicine University of Cape Town South Africa

**Keywords:** angiotensin‐1‐converting enzyme, diprolyl‐derived inhibitor, domain‐selectivity, enzyme structure, inhibitor binding, metalloprotease, X‐ray crystallography

## Abstract

Angiotensin‐1‐converting enzyme (ACE) is a zinc‐dependent carboxypeptidase of therapeutic interest for the treatment of hypertension, inflammation and fibrosis. It consists of two homologous N and C catalytic domains, nACE and cACE, respectively. Unfortunately, the current clinically available ACE inhibitors produce undesirable side effects due to the nonselective inhibition of these domains. Through structure‐based drug design, we previously identified a series of diprolyl‐derived inhibitors (SG3, SG15, SG16, SG17 and SG18) in an attempt to specifically target nACE. Only one compound, SG16, possessed significant nACEselectivity. The previously determined **16**‐nACE crystal structure (nACE:SG16) suggested interactions with Tyr369 (Phe381 in cACE) are responsible for this selectivity. To better understand the molecular basis for the lack of selectivity in the remaining compounds, we have cocrystallised nACE in complex with SG3, SG15, SG17 and SG18 and cACE in complex with SG3, SG15, SG16 and SG18 and determined their structures at high resolution. Apart from the catalytic residues, these structures further highlight the importance of residues distal to the active site that may play an important role in the design of domain‐selective inhibitors of ACE.

## Graphical abstract

Angiotensin‐1‐converting enzyme (ACE) has two catalytic domains, nACE and cACE. cACE primarily regulates blood pressure, whereas nACE is involved in fibrosis. Current inhibitors lack domain‐selectivity, thus there is a need to develop nACE and cACE‐selective inhibitors. To better understand domain‐selectivity, we determined the structures of nACE and cACE in complex with several diprolyl‐derived compounds with variable P2 moieties that utilise differences in residues within the S2 subsite.

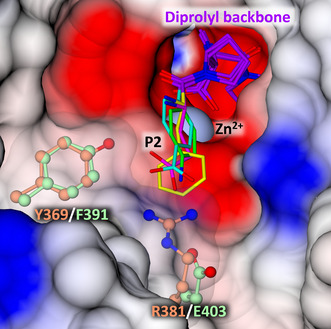

**Abbreviations**
ACE: angiotensin‐1‐converting enzyme
Ang I: angiotensin I
Ang II: angiotensin II
cACE: angiotensin‐1‐converting enzyme C‐domain
nACE: angiotensin‐1‐converting enzyme N‐domain
RAAS: renin–angiotensin–aldosterone system
sACE: somatic angiotensin‐1‐converting enzyme
tACE: testis angiotensin‐1‐converting enzyme

## Introduction

Human somatic angiotensin‐1‐converting enzyme (sACE) is a zinc‐dependent dipeptidyl carboxypeptidase involved in the regulation of blood pressure homeostasis, inflammation and immunity [1] through the hydrolysis of a range of peptides, including angiotensin I, gonadotropin hormone‐releasing hormone (GnRH), amyloid‐β, angiotensin (1–7) and *N*‐acetyl‐seryl‐aspartyl‐lysyl‐proline (AcSDKP) [2, 3, 4, 5, 6]. sACE is a type I transmembrane glycoprotein, consisting of a C‐terminal cytoplasmic region and an N‐terminal ectodomain, which are connected by a short transmembrane‐spanning region and a juxtamembrane stalk [7]. The ectodomain is a ~140 Å long dumbbell shape that is divided into two catalytic N‐ and C‐domains connected by a flexible linker [8, 9, 10]. Despite sharing 60% sequence identity and 89% sequence similarity, the N‐ and C‐domains (nACE and cACE, respectively) have different glycosylation profiles (nACE has 10 and cACE has 7 possible N‐glycosylation sites), substrate specificities, chloride dependencies and catalytic efficiencies [11, 12, 13, 14]. For example, both nACE and cACE can cleave the inactive decapeptide angiotensin I into the potent vasoconstrictor angiotensin II, but cACE does so with a threefold higher turnover compared to that of nACE [15], whereas nACE has a threefold higher turnover of AcSDKP than cACE [16]. Subsequently, cACE is the major contributor to the regulation of blood pressure [17], while nACE has roles in fibrosis and inflammation [18, 19]. The substrate bradykinin is cleaved by both domains at similar rates and causes vascular permeability and dilation [20]. This is thought to be the main mechanism by which ACE inhibitors produce side effects such as angioedema (0.1–0.7% of patients on ACE inhibitors) [21] and a population‐dependent persistent dry cough in 3.9–35% of patients [22]. The design of potent selective inhibitors of either nACE or cACE is therefore required to overcome issues related to nonselective inhibition. Currently available ACE inhibitors, such as captopril, lisinopril, enalapril and trandolapril, do not offer efficient selectivity of cACE to limit these side effects during the treatment of hypertension [23, 24]. Selective inhibitors, particularly those that specifically target nACE activity, may also broaden the application of ACE inhibitors for the treatment of cardiac and pulmonary fibrosis [25].

**Fig. 1:**
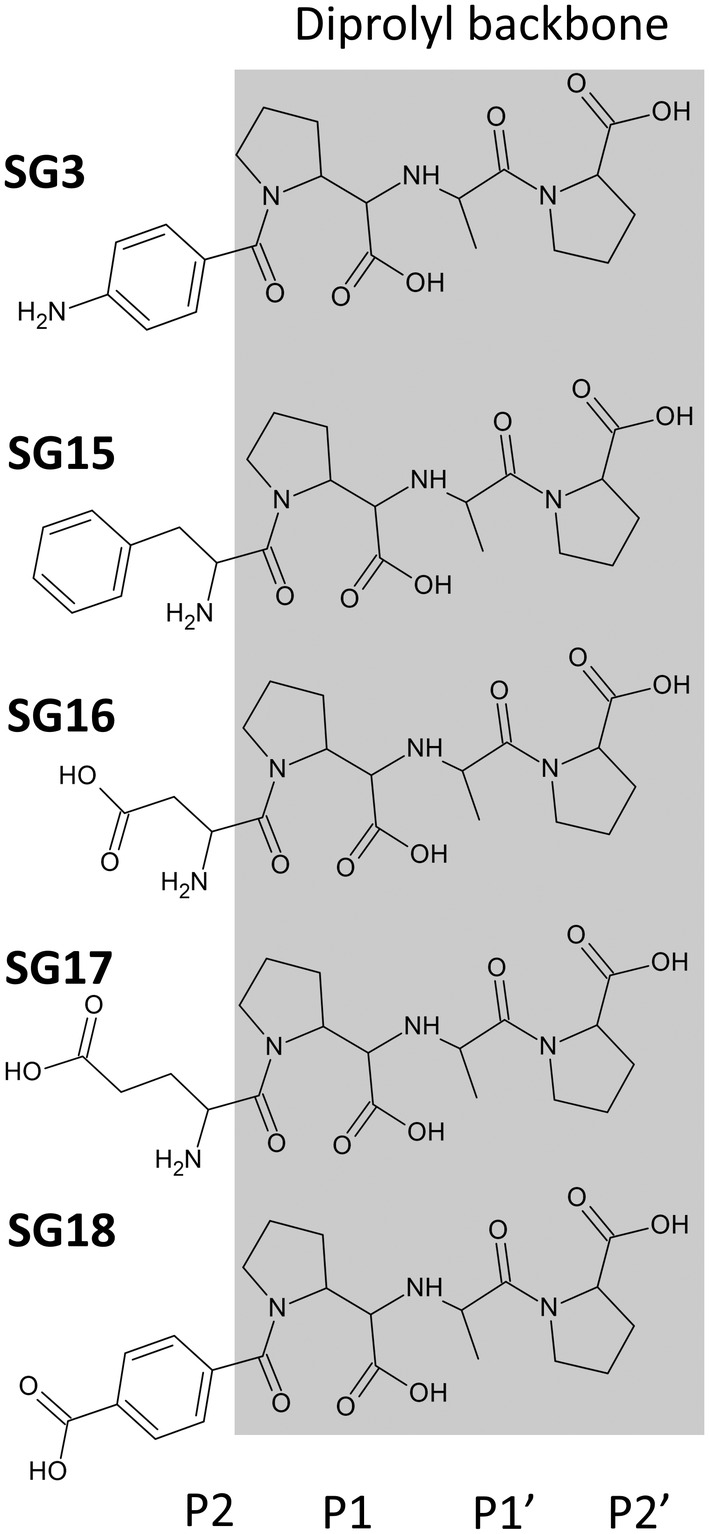
Chemical structures of diprolyl‐derived ACE inhibitors. Schechter‐Berger nomenclature is used to indicate the nonprime and prime subsites either side of the zinc‐binding carboxylic acid group. This series was synthesised with the intention of conserving binding at the S1, S1′ and S2′ subsites (grey box) but varying binding at the S2 subsite.

The first nACE‐selective inhibitor, RXP407, was identified by Dive *et al* by screening phosphonic peptide libraries. RXP407 selectively inhibits nACE by forming unique contacts with nACE‐Tyr369 (cACE‐Phe391) and nACE‐Arg381 (cACE‐Glu403). Although RXP407 possesses an nACE‐selectivity factor of 2892.34 [26, 27, 28], it has not progressed to clinical trials, as peptide inhibitors are often susceptible to proteases and have poor solubility and oral bioavailability [29]. Subsequently, the nACE‐selective inhibitor 33RE was developed with a similar binding mode to RXP407, with improved affinity for nACE but reduced selectivity [28]. In an attempt to produce an nACE‐selective inhibitor that exploits the same differences at the S2 subsite as RXP407, we previously designed a series of diprolyl‐derived compounds (SG3, SG15, SG16, SG17 and SG18) with conserved P1, P1′ and P2′ moieties, but variable P2 moieties (Fig. 1). The most promising compound, SG16, possessed an nACEselectivity factor of 83.93 and the synthesis, kinetic parameters and high‐resolution crystal structure of its complex with nACE were reported previously [30]. Additionally, SG3 and SG18 are slightly cACEselective, whereas SG15 and SG17 are slightly nACEselective (Table 1, adapted from [30]).

**Table 1:** List of *K*_*i*_ values and nACE selectivity factors for the series of diprolyl‐derived compounds and nACE and cACE‐selective inhibitors, RXP407, 33RE and RXPA380. The selectivity factor is calculated by *K*_*i*_(C)/*K*_*i*_(N). Figure was adapted from [30].

Compound	nACE (nm)	cACE (nm)	nACE selectivity factor
SG3	3.37 ± 0.55	0.22 ± 0.02	0.07
SG15	70.35 ± 9.73	167.80 ± 31.33	2.39
SG16	11.45 ± 1.37	961.02 ± 153.0	83.93
SG17	9.63 ± 1.56	17.39 ± 2.38	1.81
SG18	16.86 ± 1.24	2.29 ± 0.3	0.14
RXP407	21.03 ± 0.27	60 826 ± 175	2892.34
33RE	11.21 ± 0.735	10 395 ± 0.74	927.30
RXPA380	10000.00	3.00	0.0003

To further understand how altering the P2 moiety of diprolyl‐derived ACE inhibitors affects selectivity, we successfully cocrystallised nACE in complex with SG3, SG15, SG17 and SG18 (nACE:SG16 already reported previously, PDB codes‐ 6EN5 and 6EN6) and cACE in complex with SG3, SG15, SG16 and SG18. This information is important to consider in the future design of successful domain‐specific inhibitors.

## Results and discussion

### Crystal structures of nACE in complex with SG3, SG15, SG17 and SG18 and cACE in complex with SG3, SG15, SG16 and SG18

**Fig. 2:**
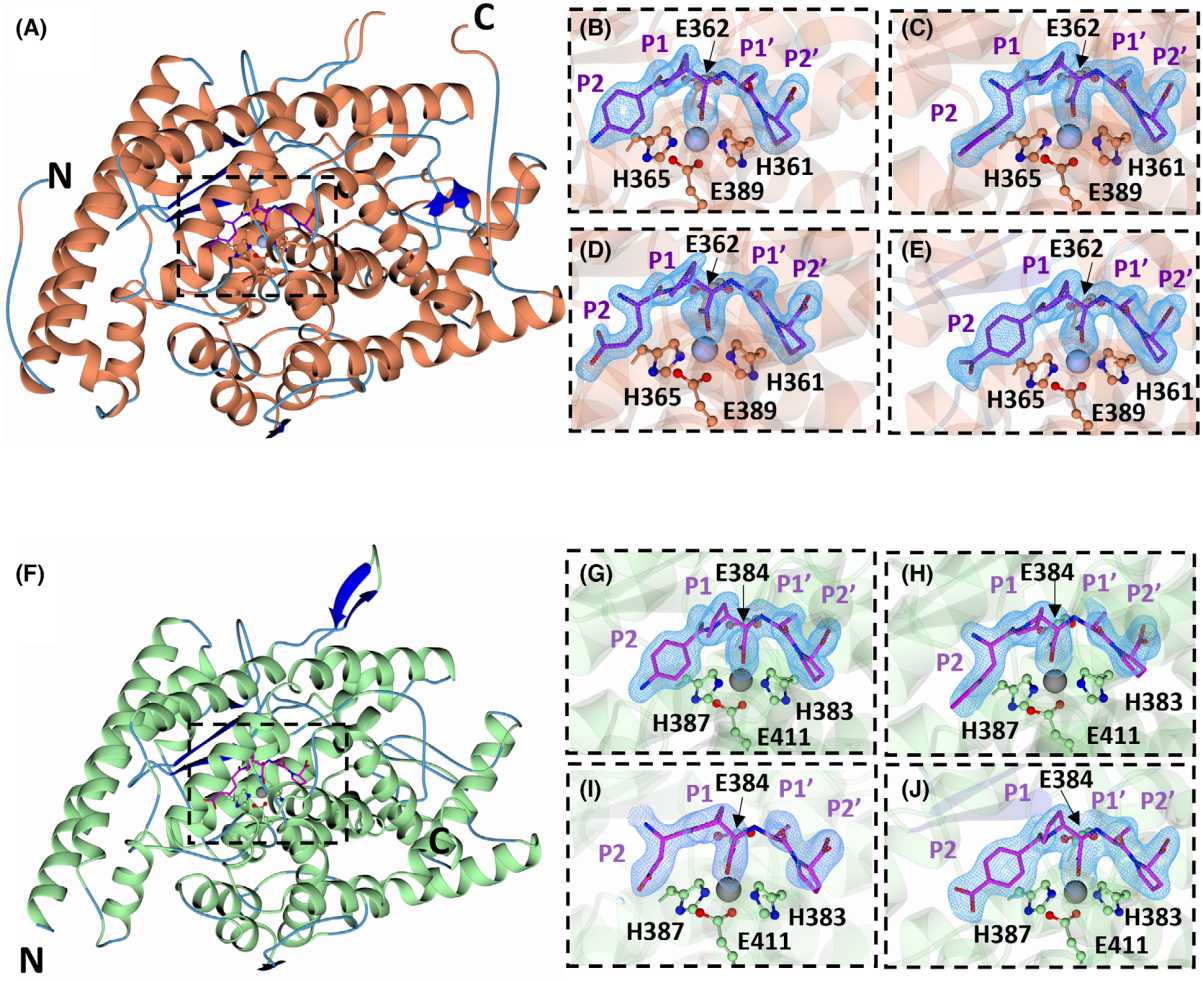
Crystal structures of nACE and cACE in complex with a series of diprolyl‐derived inhibitors. (A) Overall crystal structure of nACE (Represented by nACE:SG18 only). For nACE α‐helices are shown in orange, β‐sheets shown in blue, loops in light blue, diprolyl‐derived inhibitors in purple and zinc in silver. The binding site and unbiased difference map (Fo‐Fc, contoured at 3σ) electron density of (B) SG3, (C) SG15, (D) SG17 and (E) SG18, is shown by the blue mesh. (F) Overall crystal structure of cACE (Represented by cACE:SG18 only). For cACE, α‐helices are shown in green, β‐sheets in blue, loops in light blue, diprolyl‐derived inhibitors in magenta and zinc in grey. The binding site and unbiased difference map (Fo‐Fc, contoured at 3σ) electron density of SG3 (G), SG15 (H), SG16 (I), and SG18 (J), is shown by the blue mesh. Figure produced with CCP4MG.

The high‐resolution crystal structures of nACE:SG3, nACE:SG15, nACE:SG17, nACE:SG18, cACE:SG3, cACE:SG15, cACE:SG16 and cACE:SG18 were determined by molecular replacement using the protein chain of 6F9V (PDB) for nACE and 6F9T (PDB) for cACE as a search model. The X‐ray data collection and refinement statistics are presented in Table 2. All complexes crystallised in the typical ‘closed’ crystal form, with clear Fo‐Fc electron density at the active site corresponding to the respective bound diprolyl‐derived inhibitor (Fig. 2A–J).

**Table 2:** X‐ray data collection and refinement statistics. Outer shell statistics are shown in brackets.

Protein complex	nACE:SG3	nACE:SG15	nACE:SG17	nACE:SG18	cACE:SG3	cACE:SG15	cACE:SG16	cACE:SG18
Resolution (Å)	2.00	1.90	1.90	1.90	1.90	2.00	2.00	2.40
Space group	P1	P1	P1	P1	P 2_1_ 2_1_ 2_1_	P 2_1_ 2_1_ 2_1_	P 2_1_ 2_1_ 2_1_	P 2_1_ 2_1_ 2_1_
Cell dimensions								
*a*, *b*, *c* (Å)	72.72, 77.16, 81.84	72.5, 77.18, 81.44	73.05, 77.34, 82.91	72.72, 77.16, 81.84	55.89, 84.13, 132.66	56.36, 84.24, 133.73	56.78, 84.92, 135.01	56.82, 85.68, 135.12
α, β, γ (°)	88.37, 64.58, 75.25	88.55, 64.52, 75.14	88.38, 64.31, 74.92	88.37, 64.58, 75.25	90.00, 90.00, 90.00	90.00, 90.00, 90.00	90.00, 90.00, 90.00	90.00, 90.00, 90.00
Molecules per asymmetric unit	2	2	2	2	1	1	1	1
Completeness (%)	98.0 (96.8)	97.8 (96.1)	97.8 (96.5)	97.8 (96.4)	100.0 (100.0)	100.0 (100.0)	99.8 (99.6)	100.0 (100.0)
Rmerge	0.072 (0.473)	0.152 (5.950)	0.157 (3.039)	0.157 (3.959)	0.193 (1.584)	0.224 (1.696)	0.324 (3.208)	0.310 (1.48)
Rpim	0.046 (0.297)	0.063 (2.572)	0.054 (1.071)	0.063 (1.629)	0.057 (0.503)	0.065 (0.489)	0.066 (0.613)	0.062 (0.296)
<I/σI>	10.3 (2.5)	7.6 (0.8)	11.3 (2.6)	7.5 (1.1)	8.5 (1.5)	8.2 (1.5)	8.1 (1.4)	8.4 (2.8)
CC1/2	0.996 (0.781)	0.997 (0.394)	0.997 (0.329)	0.996 (0.433)	0.996 (0.579)	0.997 (0.559)	0.988 (0.753)	0.994 (0.841)
Multiplicity	3.5 (3.5)	6.8 (6.4)	9.5 (9.5)	7.1 (6.9)	11.9 (9.8)	12.6 (12.8)	25.9 (26.8)	25.7 (26.3)
*R*_work_/*R*_free_	0.18/0.23	0.18/0.23	0.19/0.23	0.17/0.20	0.18/0.23	0.18/0.24	0.19/0.25	0.18/0.25
RMSD bonds (Å)	0.0147	0.0130	0.0114	0.0148	0.0147	0.0129	0.0132	0.0125
RMSD angles (°)	2.463	2.596	2.260	2.400	2.251	2.583	2.545	2.672
Ramachandran Angles								
Favoured %	97.67	97.83	97.58	97.91	98.24	98.24	98.25	96.33
Allowed %	2.00	1.92	2.09	1.84	1.58	1.58	1.58	3.32
Outliers %	0.33	0.25	0.33	0.25	0.18	0.18	0.17	0.35
Average B‐factors (Å^2^)								
Protein	31.67	37.84	32.26	34.86	27.12	32.87	35.41	35.63
Ligands	45.76	55.23	50.0	56.18	48.35	60.21	65.9	67.32
Water	35.16	38.45	591	41.37	33.34	35.49	35.74	32.62
Ions	23.21	28.081	20.08	23.48	25.67	22.5	24.19	25.99
Number of nonhydrogen atoms								
Protein	9959	9855	9947	10 010	4728	4698	4756	4705
Ligands	314	289	246	385	109	150	143	125
Water	864	564	591	659	322	334	245	156
Ions	6	5	4	4	3	3	3	3
PDB code	9GBP	9GBQ	9GBR	9GBS	9GBM	9GBN	9GBO	9GBL

The nACE:SG3, nACE:SG15, nACE:SG17 and nACE:SG18 structures all crystallised with two molecules in the asymmetric unit (ASU). Superimposition of molecule A with molecule B of each structure indicates that the overall folds of the two molecules in the ASU are nearly identical, with RMSD (for Cα atoms) values in the range of 0.39–0.52 Å.

The initial Fo‐Fc electron density maps at the active site for cACE:SG15 and cACE:SG16 were noticeably less continuous in comparison to the other crystal structures, with a break in the electron density between the zinc‐binding group and the P1′ group (Fig. 2H,I). Additional unexplained density was also observed extending away from the active site towards the nonprime hole. This density is characteristic of the density observed in the previous crystal structure of cACE in which the C terminus of a symmetry‐related molecule enters through the nonprime hole (Structure cACE:C‐term, PDB code‐ 6ZPU) and coordinates the active site. Given the similarity of the C‐terminal P1, P1′ and P2′ residues that bind in the cACE:C‐term structure (PLP) to the SG compound residues (PAP), we suspect it is possible that the crystal structures of cACE:SG15 and cACE:SG16 contain a mixture of these two binding possibilities. Given the nanomolar affinities of the ligands and the presence of only partial density for the C terminus, only SG15 and SG16 have been modelled in the final deposited coordinates of cACE:SG15 and cACE:SG17. All attempts at cocrystalising cACE in complex with SG17 yielded crystal structures with the C‐terminus inserted into the active site.

### Diprolyl backbone interactions

**Fig. 3:**
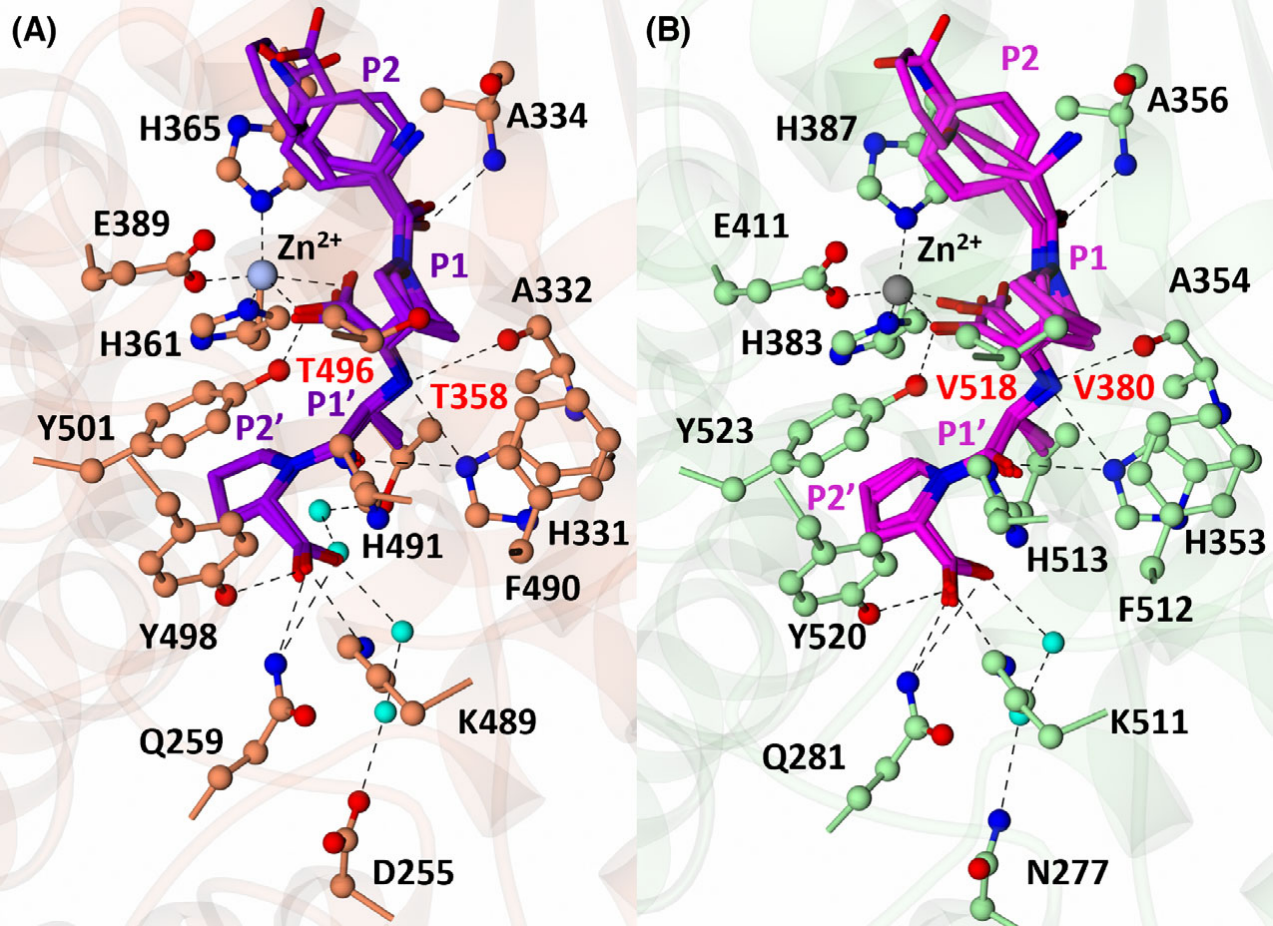
Conservation of diprolyl backbone interactions. (A) Overlay of diprolyl‐derived inhibitors from nACE:SG3, nACE:SG15, nACE:SG17 and nACE:SG18 crystal structures illustrating conservation of diprolyl backbone interactions. Only the amino acids from the nACE:SG18 structure are displayed for simplicity. The nACE amino acids are shown in orange, the diprolyl‐derived inhibitors in purple (SG3, SG15, SG17 and SG18), the zinc in silver and water molecules in cyan. Hydrogen/electrostatic interactions are shown by the dotted line. (B) Overlay of diprolyl‐derived inhibitors from cACE:SG3, cACE:SG15, cACE:SG16 and cACE:SG18. Only the amino acids from the cACE:SG18 structure are displayed for simplicity. The cACE amino acids are shown in green, the diprolyl‐derived (SG3, SG15 and SG18) inhibitors in magenta, the zinc in grey and water molecules in cyan. Hydrogen/electrostatic interactions are shown by the dotted lines. Figure produced with CCP4MG.

The diprolyl series of compounds was designed with the intention of conserving interactions at the S1, S1′ and S2′ subsites, while varying interactions at the S2 subsite, which has been identified as a driver for domain selectivity [28, 31]. The crystal structures presented here confirm that the diprolyl backbone interactions are conserved between nACE and cACE for SG3, SG15, SG16 and SG18, except for an additional two‐water‐mediated‐bridging interaction in nACE compared to in cACE (Fig. 3A,B) (Table 3). Throughout the text, the nACE residue numbering will be listed followed by the equivalent cACE residue numbering in brackets. As expected, each diprolyl‐derived inhibitor forms a coordination bond with the zinc atom through the central carboxylic acid zinc‐binding group. Interactions at the P1 position include hydrophobic contacts with nACE‐Thr496 (cACE‐Val518), nACE‐Phe490 (cACE‐Phe512) and nACE‐His331 (cACE‐His353). The P1′ alanine interacts with nACE‐Thr358 (cACE‐Val380) and nACE‐Ala332 (cACE‐Ala354) by hydrophobic contacts, with the backbone amine also forming a hydrogen bond with the carbonyl of nACE‐Ala332 (cACE‐Ala354) and the nitrogen of nACE‐His331 (cACE‐His353) and nACE‐His491 (cACE‐His513). The P2′ proline ring forms a hydrophobic interaction with nACE‐Tyr501 (cACE‐Tyr523), and the terminal carboxylic acid forms direct interactions with nACE‐Tyr498 (cACE‐Tyr520) and nACE‐Lys489 (cACE‐Lys511). In cACE, cACE‐Lys511 (nACE‐Lys489) also forms a water‐mediated interaction with the terminal carboxylic acid. Additionally, a two‐water‐mediated interaction with nACE‐Asp255 (cACE‐Asn277) is conserved between nACE and cACE, whereas nACE forms a two‐water‐mediated interaction with nACE‐Thr358 (cACE‐Val380) that is not possible in cACE. The P2 carbonyl is also conserved across the diprolyl series of compounds and forms a conserved hydrogen bonding interaction with the backbone nitrogen of nACE‐Ala334 (cACE‐Ala356).

**Table 3:** List of nACE and cACE interactions for the diprolyl‐derived compounds (SG3, SG15 SG16, SG17, SG18), nACE‐selective inhibitors (RXP407 and 33RE) and cACE‐selective inhibitor (RXPA380). Residues that form hydrogen bonds/electrostatic interactions are shown in bold, water‐mediated interactions in italics and hydrophobic/stacking interactions are underlined. The number of bridging waters between the inhibitor and residue are shown in brackets. Subsites are defined as per the Schechter and Berger nomenclature [49]. Equivalent residues are aligned and those that do not interact are shown in red for completeness.

Inhibitor	nACE				cACE			
	S2	S1	S1′	S2′	S2	S1	S1′	S2′
SG3	**A334** *D336 (2)* *Y338 (2)* H365 *Y369 (1)* *Y372 (2)* *R380 (2)* **R381** *G382 (3)* *P385 (2,3)* H388 *E389 (2)* *R500 (1)*	H331 F490 T496 R500 Y501	**H331** **A332** **H491** T358 Y501	Y122 *D255 (2)* **Q259** H331 *T358 (2)* F435 **K489** F490 H491 **Y498** Y501 F505	A356 D358 Y360 H387 F391 Y394 R402 E403 *G404 (3)* *P407 (3)* H410 *E411 (2)* R522	H353 F512 V518 R522 Y523	**H353** **A354** **H513** V380 Y523	Y146 *N277 (2)* **Q281** H353 V380 F457 ***K511** (2)* F512 H513 **Y520** Y523 F527
SG15	**A334** D336 Y338 H365 Y369 Y372 R380 R381 G382 P385 H388 E389 R500	H331 F490 T496 R500 Y501	**H331** **A332** **H491** T358 Y501	Y122 *D255 (2)* Q259 H331 *T358 (2)* F435 **K489** F490 H491 **Y498** Y501 F505	**A356** D358 Y360 H387 F391 Y394 R402 E403 G404 P407 H410 E411 R522	H353 F512 V518 R522 Y523	**H353** **A354** **H513** V380 Y523	Y146 *N277 (2)* Q281 H353 V380 F457 ***K511**(2)* F512 H513 **Y520** Y523 F527
SG16	**A334** D336 Y338 H365 **Y369** Y372 R380 *R381 (1)* G382 P385 H388 *E389 (2)* **R500**	H331 F490 T496 R500 Y501	**H331** **A332** **H491** T358 Y501	Y122 *D255 (2)* **Q259** H331 *T358 (2)* F435 **K489** F490 H491 **Y498** Y501 F505	**A356** D358 Y360 H387 F391 Y394 R402 E403 G404 P407 H410 E411 **R522**	H353 F512 V518 R522 Y523	**H353** **A354** **H513** V380 Y523	Y146 *N277 (2)* **Q281** H353 V380 F457 ***K511**(2)* F512 H513 **Y520** Y523 F527
SG17	**A334** D336 Y338 H365 **Y369** Y372 R380 **R381** *G382 (3)* *P385 (2,3)* H388 *E389 (2)* **R500**	H331 F490 T496 R500 Y501	**H331** **A332** **H491** T358 Y501	Y122 *D255 (2)* **Q259** H331 *T358 (2)* F435 **K489** F490 H491 **Y498** Y501 F505	Not determined	Not determined	Not determined	Not determined
SG18	**A334** *D336 (2,2)* *Y338 (1)* H365 ***Y369*** Y372 R380 **R381** *G382 (3)* *P385 (2,3)* H388 *E389 (2)* *R500 (1)*	H331 F490 T496 R500 Y501	**H331** **A332** T358 **H491** Y501	Y122 *D255 (2)* **Q259** H331 *T358 (2)* F435 **K489** F490 H491 **Y498** Y501 F505	*A356 (2)* *D358 (2)* *Y360 (1)* H387 F391 *Y394 (2)* R402 E403 G404 P407 H410 E411 **R522**	H353 F512 V518 R522 Y523	**H353** **A354** V380 **H513** Y523	Y146 *N277 (2)* **Q281** H353 V380 F457 ***K511** (2)* F512 H513 **Y520** Y523 F527
RXP407	**A334** D336 Y338 H365 **Y369** Y372 R380 **R381** G382 P385 H388 E389 R500	H331 F490 T496 R500 Y501	**H331** **A332** **H491** T358 Y501	Y122 *D255 (2)* **Q259** H331 T358 F435 K489 F490 **H491** **Y498** Y501 F505	Not determined	Not determined	Not determined	Not determined
33RE	***A334 (2)*** *D336 (2)* *Y338 (2)* H365 ***Y369 (2)*** *Y372 (2)* *R380 (2)* R381 G382 *P385 (1)* H388 *E389 (1)* R500	H331 F490 T496 *R500 (1)* *Y501 (1)*	**H331** A332 **H491** T358 Y501	*Y122 (2)* *D255 (2)* **Q259** H331 T358 F435 **K489** F490 H491 **Y498** Y501 F505	Not determined	Not determined	Not determined	Not determined
RXPA380	Not determined	Not determined	Not determined	Not determined	**A356** D358 Y360 H387 F391 Y394 R402 E403 G404 P407 H410 E411 R522	H353 F512 V518 R522 Y523	**H353** A354 V380 **H513** **Y523**	*Y146 (2)* N277 **Q281** H353 V380 F457 **K511** F512 H513 **Y520** Y523 F527

### S2 subsite interactions of nACE and cACE in complex with SG3

**Fig. 4:**
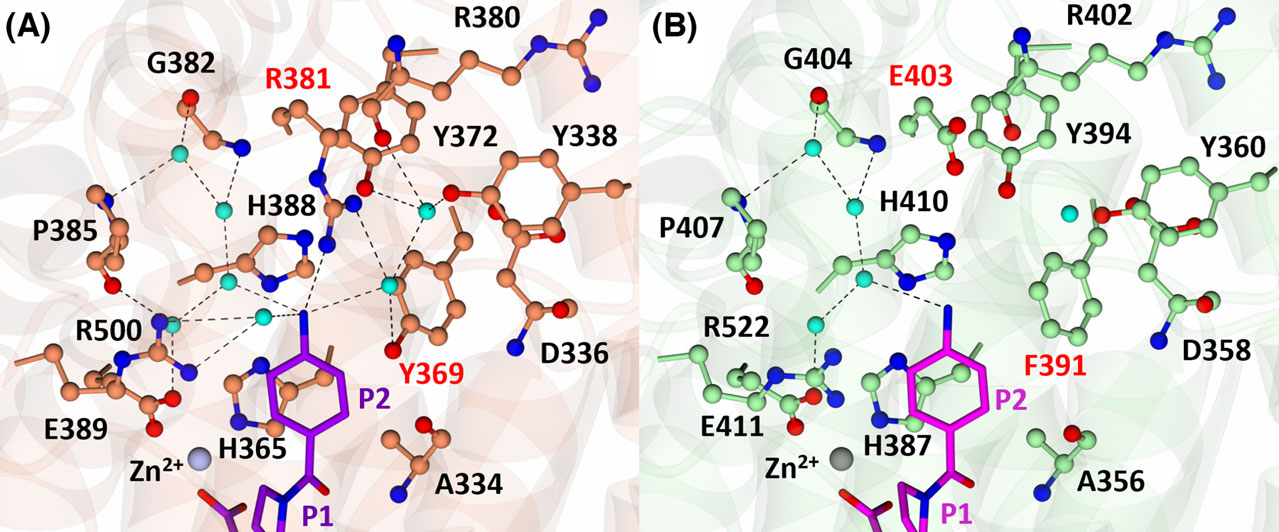
Interactions of SG3 with nACE (left) and cACE (right) at the S2 subsite. (A) S2 subsite of nACE in complex with SG3. Residues of nACE are shown in orange and SG3 in purple. The zinc atom is shown as a silver sphere and water molecules in cyan. (B) S2 subsite of cACE in complex with SG3. Residues of cACE are shown in green and SG3 in magenta. The zinc atom is shown as a grey sphere and water molecules in cyan. Hydrogen/electrostatic interactions are shown by the dotted lines. Figure produced with CCP4MG.

In both nACE and cACE, the P2 aniline ring of SG3 (Fig. 1) stacks with nACE‐His365 (cACE‐His387). Additionally, a network of four water molecules bridge residues nACE‐Gly382 (cACE‐Gly404), nACE‐Pro385 (cACE‐Pro407) and nACE‐Glu389 (cACE‐Glu411) to the aniline nitrogen of SG3 (Fig. 4A,B). In nACE, there is a two‐water‐mediated interaction with nACE‐Asp336, nACE‐Tyr372, nACE‐Tyr338 and the carbonyl of nACE‐Arg380 (Fig. 4A), which is not present in cACE due to substitutions of nACE‐Arg381 and nACE‐Tyr369 with cACE‐Glu403 and cACE‐Phe391, respectively (Fig. 4B). These substitutions result in the inability of cACE to coordinate an equivalent bridging water molecule with SG3. Additionally, in nACE, nACE‐Arg381 (cACE‐Glu403) forms a direct hydrogen bond with SG3; however, this is likely to be a weak and transient interaction due to the distance of the hydrogen bond in molecule A (3.53 Å) and its absence in molecule B (due to insufficient density to accurately model cACE‐Arg381 in molecule B). Interestingly, despite SG3 forming seven fewer polar interactions with cACE, cACE:SG3 has a lower *K*_i_ value than nACE:SG3 (Table 1). This is likely due to the substitution of cACE‐Phe391 with nACE‐Tyr369, where in cACE the aniline ring is stabilised by the more hydrophobic S2 subsite, but in nACE it is destabilised due to the additional hydroxyl group (Fig. 4A,B). This destabilisation in nACE is further evidenced by a comparison of the B‐factors of the P2 ring atoms to the P2 carbonyl carbon atom. The position and binding of the P2 carbonyl is conserved across all the structures presented here and therefore provides a reference point for comparison of B‐factors. In nACE:SG3, the P2 ring atoms have B‐factors larger than its respective P2 carbonyl carbons, whereas in cACE:SG3 they are smaller (Table 3) indicating greater stability of the P2 ring in cACE.

### S2 subsite interactions of nACE and cACE in complex with SG15

**Fig. 5:**
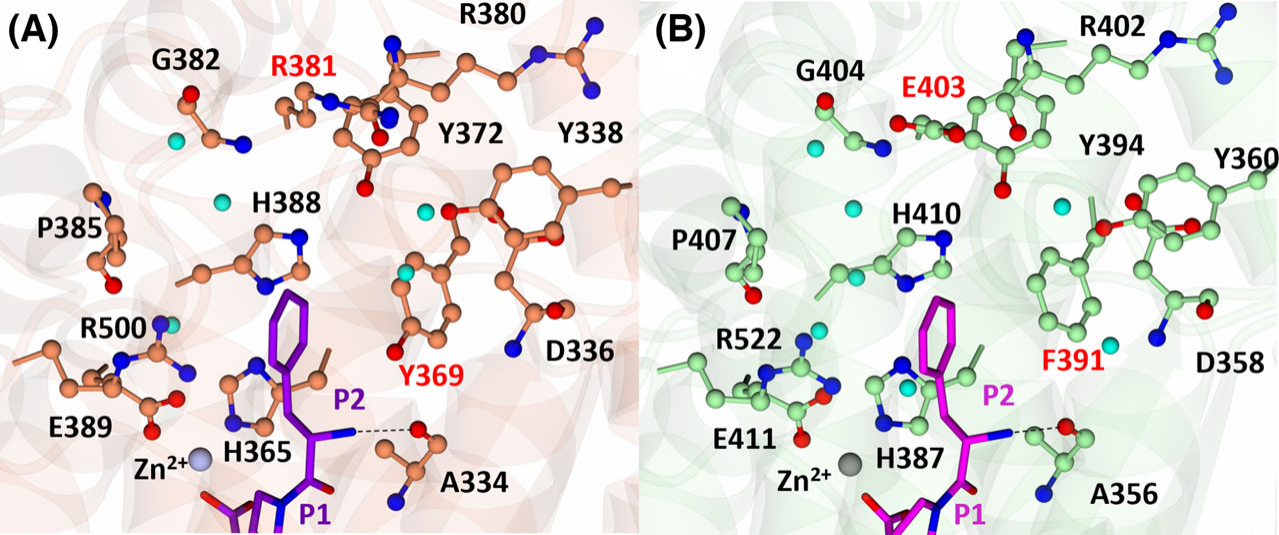
Interactions of SG15 with nACE (left) and cACE (right) at the S2 subsite. (A) S2 subsite of nACE in complex with SG15. Residues of nACE are shown in orange and SG15 in purple. The zinc atom is shown as a silver sphere and water molecules in cyan. (B) S2 subsite of cACE in complex with SG15. Residues of cACE are shown in green and SG15 in magenta. The zinc atom is shown as a grey sphere and water molecules in cyan. Hydrogen/electrostatic interactions are shown by the dotted lines. Figure produced with CCP4MG.

A comparison of nACE:SG15 and cACE:SG15 indicates almost identical binding of the SG15 P2 moiety (Fig. 1) in both nACE and cACE (Fig. 5A,B). The orientation of the ring is conserved, where it forms a hydrophobic stacking interaction with nACE‐His388 (cACE‐His410), and the backbone forms a hydrogen bond with the carbonyl oxygen of nACE‐Ala334 (cACE‐Ala356) via its amine group. However, in nACE there appears to be disruption of four water molecules that contribute to stabilisation of nACE‐Gly382 (cACE‐Gly404), nACE‐Pro385 (cACE‐Pro401) and nACE‐Glu389 (cACE‐Glu411) residues, which is observed in nACE:SG3 and cACE:SG3 (Table 3). This disruption is not evident in the cACE:SG15 structure. Surprisingly, despite cACE containing a more hydrophobic S2 subsite, the P2 ring appears to be more stable in nACE:SG15 based on a comparison of P2 ring's atomic B‐factors (Table 4), which may explain the increased affinity for nACE.

**Table 4:** List of B‐factors for SG compounds containing a P2 ring (molecule A only).

Complex	P2 carbonyl carbon (Å^2^)	Cγ (Å^2^)	Cδ1 (Å^2^)	Cδ2 (Å^2^)	Cε1 (Å^2^)	Cε2 (Å^2^)	Cζ (Å^2^)
nACE:SG3	18.11	21.45 (3.34)	24.15 (6.04)	21.28 (3.17)	24.49 (6.38)	25.49 (7.38)	27.52 (9.41)
cACE:SG3	19.80	19.10 (−0.70)	17.77 (−2.03)	18.94 (−0.86)	18.88 (−0.92)	19.04 (−0.76)	19.10 (−0.70))
nACE:SG15	23.82	28.77 (4.95)	30.63 (6.81)	44.81 (20.99)	38.65 (14.83)	48.82 (25.00)	40.35 (16.53)
cACE:SG15	22.20	31.17 (8.97)	33.77 (11.57)	40.43 (18.23)	40.20 (18.00)	47.67 (25.47)	51.92 (29.72)
nACE:SG18	27.17	27.91 (0.74)	28.25 (1.08)	27.14 (−0.03)	27.13 (−0.04)	29.06 (1.89)	28.14 (0.97)
cACE:SG18	25.96	23.64 (−2.32)	24.92 (−1.04)	22.66 (−3.3)	24.92 (−1.04)	21.56 (−4.4)	30.54 (4.58)

Atoms are labelled according to amino acid labelling. A comparison of the B‐factor of the P2 carbonyl carbon to those of the atoms within the ring highlights the difference in stability of P2 ring moieties across the diprolyl series. The difference, relative to the P2 carbonyl is shown in brackets.

### S2 subsite interactions of nACE and cACE in complex with SG16

**Fig. 6:**
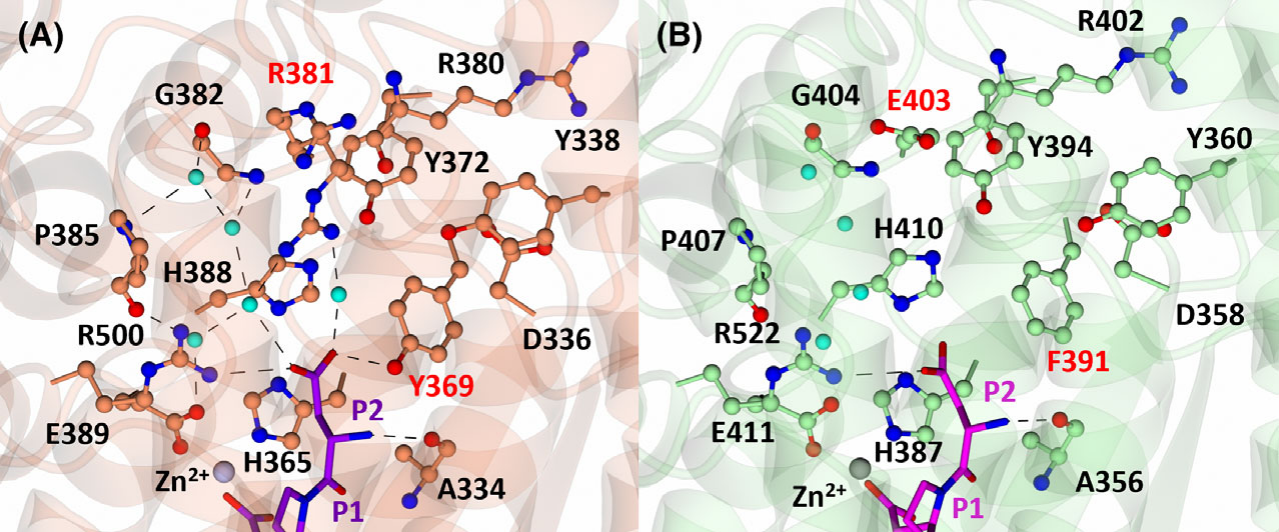
Interactions of SG16 with nACE (left) and cACE (right) at the S2 subsite. (A) S2 subsite of nACE in complex with SG16 (PDB codes‐6EN5 and 6EN6). Residues of nACE are shown in orange and SG16 in purple. The zinc atom is shown as a silver sphere and water molecules in cyan. (B) S2 subsite of cACE in complex with SG16. Residues of cACE are shown in green and SG16 in magenta. The zinc atom is shown as a grey sphere and water molecules in cyan. Hydrogen/electrostatic interactions are shown by the dotted lines. Figure produced with CCP4MG.

The interacting residues of nACE:SG16 at the S2 subsite were previously described, where the P2 carboxylic acid group (Asp mimic) (Fig. 1) interacts with nACE‐Tyr369, nACE‐Arg381, nACE‐Arg500 and the P2 amine group with nACE‐Ala334 (Fig. 6A, Table 3). In cACE, nACE‐Arg381 and nACE‐Tyr369 are substituted by cACE‐Phe391 and cACE‐Glu403 and thus do not interact; however, the interaction with cACE‐Arg522 (nACE‐Arg500) is conserved (Fig. 6B). In the case of SG16, loss of these interactions in cACE appears to contribute significantly to the loss of inhibitor potency (Table 1); however, as observed for the other inhibitors in the series, loss of direct interactions does not always correlate to a reduced affinity.

### S2 subsite interactions of nACE in complex with SG17 and potential cACE interactions

**Fig. 7:**
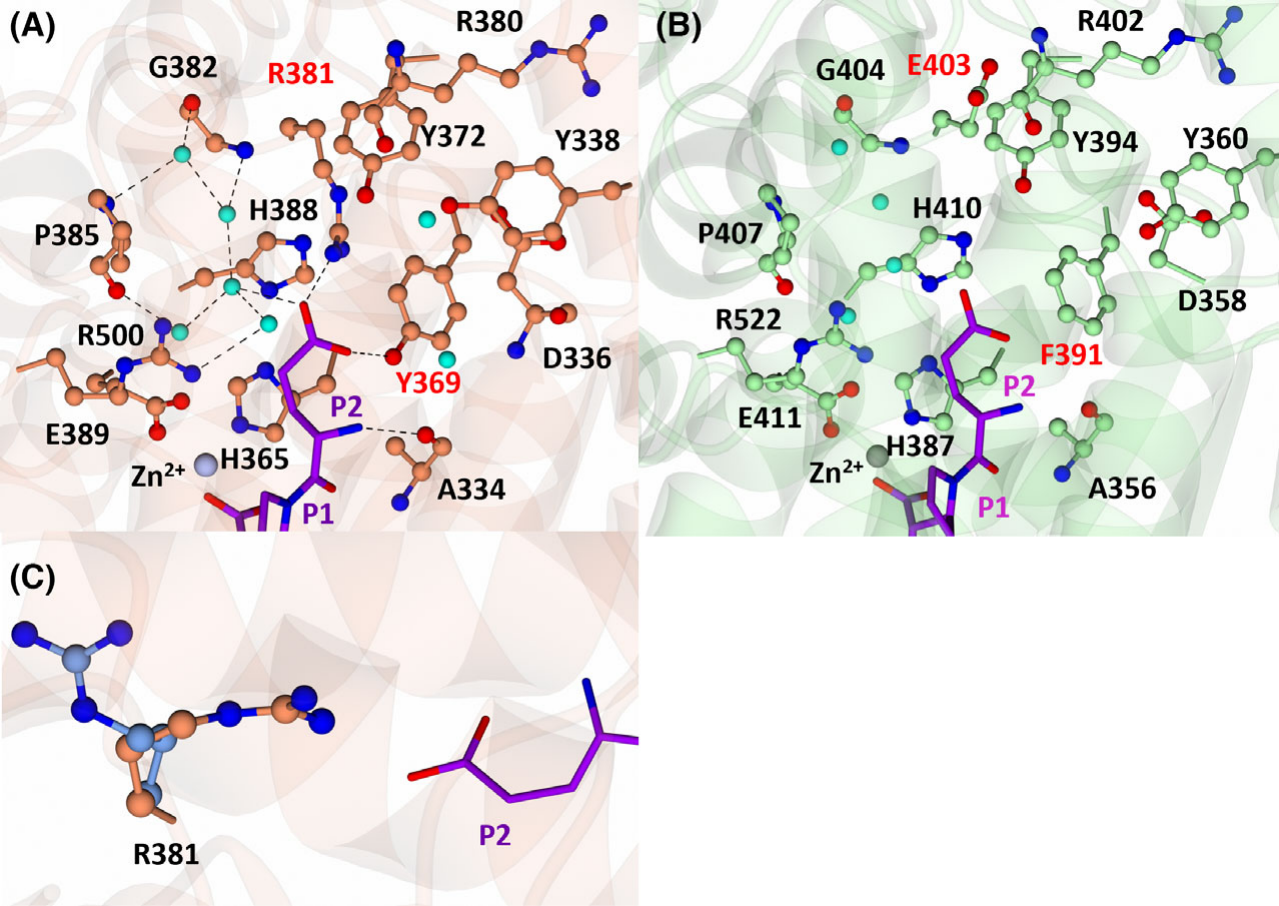
Interactions of SG17 with nACE (left) and superimposition of nACE:SG17 with the native structure of cACE (right) at the S2 subsite. (A) S2 subsite of nACE in complex with SG17. Residues of nACE are shown in orange and SG17 in purple. The zinc atom is shown as a silver sphere and water molecules in cyan. (B) Superimposition of nACE:SG17 with native cACE (PDB code 1O8A) indicating how SG17 might bind cACE. The nACE residues are omitted for clarity. Residues of cACE are shown in green. The zinc atom is shown as a grey sphere and water molecules in cyan. (C) Different conformations of R381 in nACE:SG18, molecule A is shown in orange and molecule B in light blue. Hydrogen/electrostatic interactions are shown by the dotted lines. Figure produced with CCP4MG.

In the nACE:SG17 structure, the P2 carboxylic acid group of SG17 (Glu mimic) (Fig. 1) forms a hydrogen bond and salt‐bridge interaction with nACE‐Tyr369 and nACE‐Arg381, respectively, which are not feasible in cACE due to substitution of these residues (cACE‐Phe391 and cACE‐Glu403). SG17 is further stabilised by a network of four water molecules connecting the carboxylic acid group to residues nACE‐Gly382 (cACE‐Gly404), nACE‐Pro385 (cACE‐Pro407) and nACE‐Glu389 (cACE‐411). Additionally, the backbone P2 amine forms a hydrogen bond with nACE‐Ala334 (cACE‐356) (Fig. 7A). Attempts at cocrystalising cACE in complex with SG17 only yielded crystal structures where the C terminus of a symmetry‐related molecule had inserted into the active site (previously described by Cozier *et al*. [32]). In order to determine the potential interactions of cACE with SG17, we superimposed the structure of nACE:SG17 with the native cACE structure (PDB code 1O8A) (Fig. 7B). This analysis, along with the analysis of the cACE:SG16 crystal structure, suggests SG17 would bind in a similar way, except that the carboxylic acid would likely be positioned towards cACE‐Arg522 due to the nACE‐Tyr369 and nACE‐Arg381 substitutions (cACE‐Phe391 and cACE‐Glu403, respectively). Interestingly, nACE‐Arg381, the residue that the P2 glutamic acid moiety was designed to target, only forms an interaction in molecule A. In molecule B, nACE‐Arg381 is positioned away from this moiety and does not form an interaction (Fig. 7C). This weak interaction with nACE‐Arg381 was also observed in the crystal structure of nACE:SG16 (PDB codes 6EN5 and 6EN6). However, in nACE:SG16, the interaction is through a water molecule, whereas in SG17 it is via a direct hydrogen bond/salt‐bridge interaction. This suggests that the targeting of nACE‐Arg381 by the P2 glutamic acid/aspartic acid moiety is not strong and is weaker in nACE:SG16 due to solvation effects.

### S2 subsite interactions of nACE and cACE in complex with SG18

**Fig. 8:**
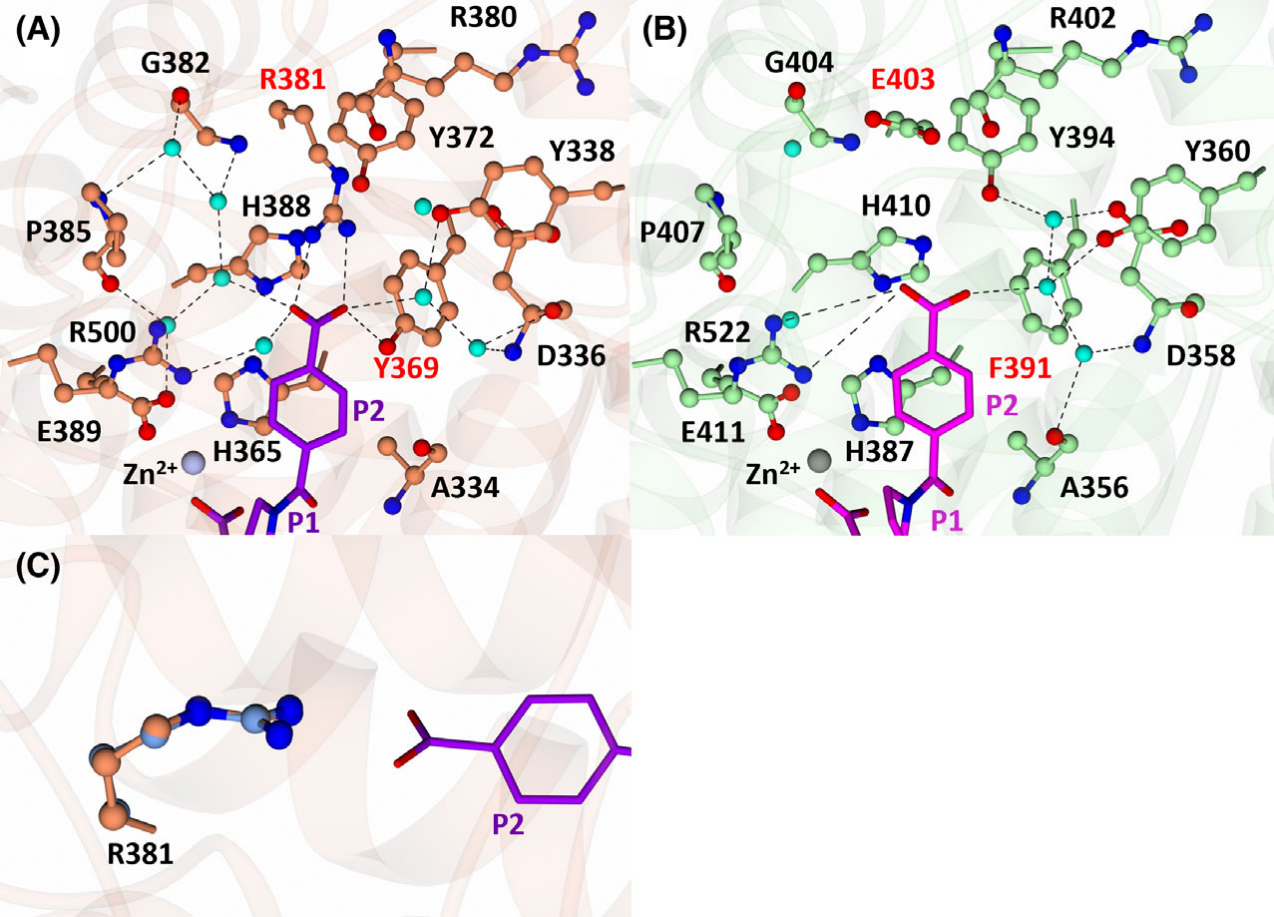
Interactions of SG18 with nACE (left) and cACE (right) at the S2 subsite. (A) S2 subsite of nACE in complex with SG18. Residues of nACE are shown in orange and SG18 in purple. The zinc atom is shown as a silver sphere and water molecules in cyan. (B) S2 subsite of cACE in complex with SG18. Residues of cACE are shown in green and SG18 in magenta. The zinc atom is shown as a grey sphere and water molecules in cyan. (C) Conformations of R381 in nACE:SG18, molecule A is shown in orange and molecule B in light blue. Hydrogen/electrostatic interactions are shown by the dotted lines. Figure produced with CCP4MG.

The P2 moiety of SG18 is composed of a six‐membered ring with a terminal carboxylic acid (Fig. 1). In both nACE and cACE, the P2 ring of SG18 interacts with nACE‐His365 (cACE‐His387) via a π‐stacking interaction and with nACE‐Ala334 (cACE‐Ala356) via hydrophobic contacts. Most of the other interactions are through the terminal carboxylic acid group. In nACE, the P2 carboxylic acid forms a hydrogen bond with the hydroxyl group of nACE‐Tyr369 (cACE‐Phe391) and a salt bridge with nACE‐Arg381 (cACE‐Glu403), which are not possible in cACE (Fig. 8A,B). In both nACE and cACE, the carboxylic acid of SG18 makes contact with nACE‐Tyr338 (cACE‐Tyr360) via a single water molecule and with nACE‐Asp336 (cACE‐Asp358) via two water molecules (Fig. 8A,B). However, in cACE, there is also a water‐mediated bridge to cACE‐Tyr394 and the side chain of cACE‐Asp358 (Fig. 8B). SG18 also interacts with nACE and cACE via nACE‐Arg500 and cACE‐Arg522, respectively. In nACE, this interaction is through a water molecule, while in cACE it is via a long‐range electrostatic interaction.

Despite the presence of a carboxylic acid within the P2 moiety of SG18 that was designed to selectivity target the nACE‐specific residues nACE‐Arg381 (cACE‐Glu403) and nACE‐Tyr369 (cACE‐Phe391), this inhibitor binds cACE more strongly (Table 1). In cACE, the instability that would be caused by the presence of the carboxylic acid close to cACE‐Phe391 within the S2 subsite, and the inability to interact with cACE‐Glu403, is off‐set by the presence of the P2 ring, which makes hydrophobic contacts with cACE‐Phe391. This results in the repositioning of the carboxylic acid away from cACE‐Phe391, where it is stabilised by three water molecules. Conversely, the hydroxyl group of nACE‐Tyr369 causes instability within the P2 ring of nACE:SG18, which is evidenced by comparing the B‐factors of the P2 ring with the B‐factors of the P2 carbonyl carbon (which, as previously stated, facilitates a comparison of B‐factors across varying resolutions). In nACE:SG18, the B‐factors of the P2 ring are larger than in the P2 carbonyl carbon, whereas in cACE:SG18 they are lower (Table 3) indicating greater stability of the P2 ring in cACE.

### Arg522 forms a cation‐H_2_O‐π interaction with the P2 ring in cACE:SG3

**Fig. 9:**
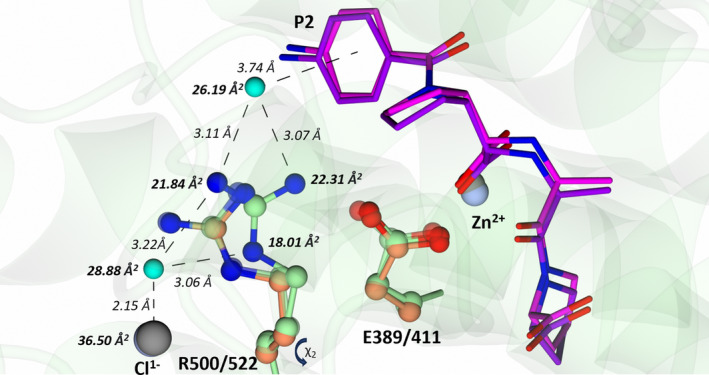
cACE:SG3 Cation‐H_2_O‐π interaction. cACE in complex with SG3 forms a cation‐H_2_O‐π interaction between cACE‐Arg522 and the P2 ring of SG3. This results in the displacement of cACE‐Arg522 by ~1 Å, facilitating the coordination of an additional water molecule to the conserved chloride and destabilisation of active site by disruption of cACE‐Glu411. nACE:SG3 residues are shown in orange, cACE:SG18 in transparent green, cACE:SG3 in opaque green, SG3 from nACE:SG3 in purple and cACE:SG3 in magenta. Both the zinc and chloride ions, for nACE and cACE, are shown by silver and grey spheres, respectively. B‐factors (bold) and bond distances (*italics*) for interacting atoms are shown for the cACE:SG3 structure only. Only nACE:SG3, cACE:SG3 and cACE:SG18 are shown for simplicity. The R500/522 residue of the nACE:SG3, nACE:SG15, nACE:SG17 and cACE:SG15 structures adopt a similar position to nACE:SG3 and cACE:SG18. Hydrogen/electrostatic interactions are shown by the dotted lines. Figure produced with CCP4MG.

A comparison of cACE‐Arg522 in the cACE:SG3 structure to cACE‐Arg522 in all nACE (nACE‐Arg500) and cACE structures revealed a striking difference in the cACE‐Arg522 position of cACE:SG3, whereby cACE‐Arg522 was rotated by 13.54° about *χ* ^2^ (Fig. 9). Upon binding SG3, the Cζ of cACE:SG3 shifted by an average of ~1.0 Å away from the conserved chloride that coordinates nACE‐Arg500 (cACE‐Arg522) in all nACE and cACE structures to‐date. This exposes the chloride ion to the solvent and facilitates the binding of an additional water molecule which, along with the shift of cACE‐Arg522, destabilises the coordination of the chloride ion due to solvation effects. The repositioning of cACE‐Arg522 also results in a cation‐H_2_O‐π interaction with the P2 ring of SG3 (evidenced by an off‐set distance of 3.74 Å from the ring centroid [33, 34]), and an electrostatic interaction with the zinc ion coordinating cACE‐Glu411 (Fig. 9). An equivalent water to the cation‐H_2_O‐π water is also present in the nACE:SG3, nACE:SG18 and cACE:SG18 structures, which contain a P2 ring. However, nACE‐Arg500 (cACE‐Arg522) has not repositioned to form a strong bidentate interaction with the water as seen in cACE:SG3. Interestingly, despite possessing a P2 ring, SG15 does not contain the cation‐H_2_O‐π interaction and is the weakest inhibitor of the series. Given the chloride ion dependency of ACE, more so for cACE, the observed dynamics of nACE‐Arg500 (cACE‐Arg522) may be involved in exposing the chloride ion to the catalytic cleft and substrates or inhibitors that extend beyond the S2 subsite. Previous experiments have shown that chloride ion binding influences the binding strength of different substrates and inhibitors through chloride‐mediated enhancement [12, 35]. In the native cACE structures, the coordination of cACE‐Arg522 to the chloride ion prevents a hydrogen bond between cACE‐Arg522 and cACE‐Glu411 (a conserved active site residue) that disrupts key active site residues [36].

### nACE‐Arg381 does not provide strong direct interactions that drive nACE selectivity

**Fig. 10:**
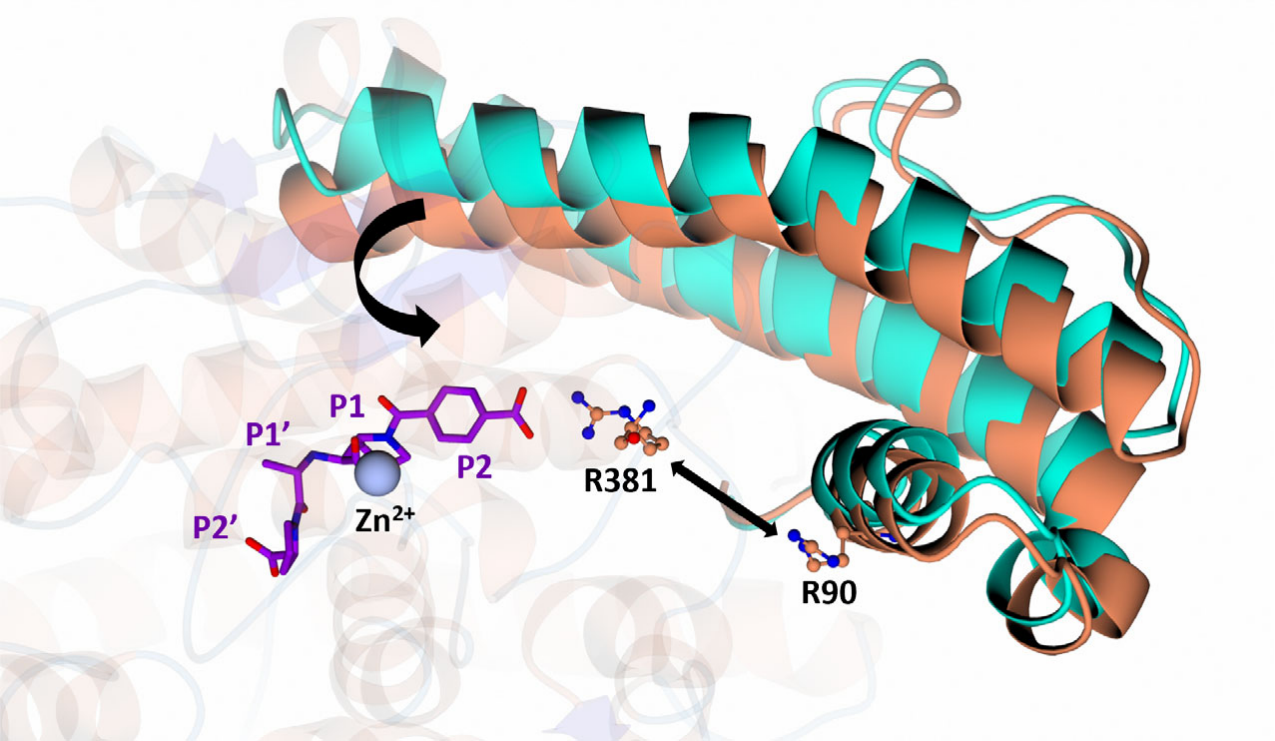
Involvement of nACE‐Arg90 and nACE‐Arg381 in cleft closure. It has been proposed that repulsion of nACE‐Arg90 by nACE‐Arg381 favours cleft closure by tilting of the lid‐region towards the active site. Anchoring of nACE‐Arg381 by SG18 may therefore hinder cleft closure as it ‘locks’ the conformation of nACE‐Arg381 away from nACE‐Arg90. The lid‐region of nACE:SG18 is shown in orange and the nACE open structure (PDB code 6ZPQ) in cyan indicating how the lid‐region opens and closes. SG18 is shown in purple. The bent arrow represents the tilting motion from open (cyan) to closed (orange). The straight arrow represents repulsion of nACE‐Arg90 by nACE‐Arg381. Figure produced with CCP4MG.

In the previous structures of nACE in complex with SG16 (PDB codes 6EN5 and 6EN6), nACE‐Arg381 only forms a water‐mediated interaction with SG16 in ~30% of the conformations observed. The remaining ~70% adopt different conformations away from SG16 [30], suggesting nACE‐Arg381 is highly flexible. The presence of two molecules within the ASU of the nACE:SG3, nACE:SG15, nACE:SG17 and nACE:SG18 structures further reveals a high degree of flexibility of nACE‐Arg381, even upon binding of strong inhibitors designed to target this residue (SG16, SG17 and SG18). As discussed above, for nACE:SG17, the carboxylic acid of SG17 forms a salt bridge with nACE‐Arg381 in molecule A, but not in molecule B. In the nACE:SG18 structure, nACE‐Arg381 interacts with the carboxylic acid group in both molecules of the ASU and possesses low B‐factors, indicative of a stable interaction. Therefore, flexibility of nACE‐Arg381 may partially explain its inefficiency at forming an interaction with carboxylic acid moieties within the S2 subsite (Figs 7C and 8C). Without designing inhibitors that extend beyond the S2 pocket, nACE‐Arg381 appears to be a poor residue to target in the design of nACE‐selective inhibitors. In fact, targeting of nACE‐Arg381 may contribute to a reduction in ligand affinity, due to the involvement of nACE‐Arg381 and nACE‐Arg90 in cleft closure. It was previously suggested that the location of nACE‐Arg381 within a flexible loop close to nACE‐Arg90 drives the tipping motion of the lid‐region through repulsion of nACE‐Arg90 by nACE‐Arg381 [37, 38]. Thus, anchoring nACE‐Arg381 away from nACE‐Arg90 may reduce binding strength by hindering cleft closure (Fig. 10). This, in part, may contribute to the reduced affinity nACE has for SG18 (compared to SG16 and SG17), despite its stable interaction with nACE‐Arg381 and why, although cACE possesses fewer interactions with SG3 and SG18, it has a higher affinity than nACE (Table 1). The difference in binding affinity when compared with nACE:SG18 and cACE:SG18 could also be explained by the instability caused by the position of the SG18 P2 ring close to the hydroxyl of nACE‐Tyr369. This is also observed for SG3, which is identical to SG18 except the carboxylic acid group designed to target nACE‐Arg381 is replaced with an amine group and is therefore unable to form a salt bridge with nACE‐Arg381. Despite this, SG3 is not a weaker nACE binder than SG18, further emphasising the negative effect on binding affinity by anchoring nACE‐Arg381 away from nACE‐Arg90. In contrast, SG15 is incapable of forming any interaction with nACE‐Arg381 and is the weakest binder of nACE in the series. This may be explained by the instability of the P2 ring within the charged S2 subsite of nACE. Despite this, nACE‐Arg381 may have separate effects to that of direct ligand binding which contributes to the differing *K*_*i*_ values (Table 1) observed for the SG compounds. In particular, previous mutagenesis studies that mutated nACE residues within the S2 and S′ subsites to their cACE counterparts (S2‐nACE [Y369F and R381E] and S2‐S′‐nACE [Y369F, R381E, S260E, E262S, D354E, S357V and E431D]) had varying effects on the affinity of the nACE‐selective inhibitors 33RE, SG16 and KetoACE‐13, as well as the partially cACE‐selective enalaprilat [37, 38]. For 33RE, SG16 and KetoACE‐13, the S2 and S′ mutations independently reduced the affinity and caused a pronounced reduction when combined. However, for KetoACE‐13, S2‐nACE had ~2‐fold lower affinity than cACE, and surprisingly, for enalaprilat all mutants had reduced affinity [37]. Together, this indicates a property distal to the cACE active site is able to compensate for the loss of interactions with nACE‐Tyr369 (cACE‐Phe391) and nACE‐Arg381 (cACE‐Glu403) and may explain why SG3 and SG18 are better binders for cACE, despite possessing fewer direct interactions.

### Comparison with nACE‐specific inhibitors

**Fig. 11:**
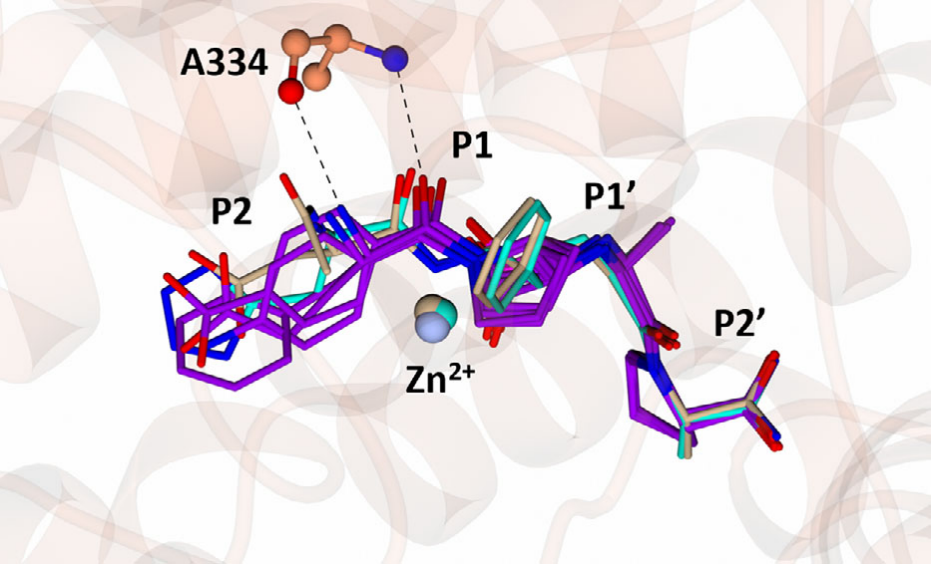
Comparison of nACE in complex with SG3, S15, SG17, SG18, RXP407 and 33RE. Superimposition of nACE:SG3, nACE:SG15, nACE:SG17, nACE:SG18, nACE:RXP407 (PDB code 3NXQ) and nACE:33RE (PDB code 4BXK) reveal similar positioning of the inhibitors within the active site, except for the P2 moiety. SG compounds are shown in purple, RXP407 in beige and 33RE in cyan. The zinc atoms from the nACE structures in complex with SG compounds are shown in silver, RXP407 in beige and 33RE in cyan. Only nACE‐Ala334 of nACE:SG15 is shown for simplicity. Hydrogen bonds are shown by the dotted lines. Figure produced with CCP4MG.

The diprolyl‐derived inhibitors presented here, and previously, possess dissociation constants within the nanomolar range, similar to what has been observed in the nACE‐specific inhibitors RXP407 and 33RE (Table 1). However, with the exception of SG16, these inhibitors lack the desired nACE selectivity achieved by RXP407 and 33RE. Superimposition of nACE:SG3, nACE:SG15, nACE:SG17 and nACE:SG18, with nACE:RXP407 [39] (PDB code 3NXQ) and nACE:33RE [28] (PDB code 4BXK) show that these compounds occupy a similar position (Fig. 11), with the interactions at the S2, S1, S1′ and S2′ subsites mostly conserved despite variations of the P2 moiety (Table 3). SG3, SG17 and SG18 retain interactions to the nACE‐specific residues nACE‐Arg381 and nACE‐Tyr369. However, for SG3 and SG17, the interaction to nACE‐Arg381 is likely weak due to its transient nature. On the other hand, SG18 forms a strong interaction with nACE‐Arg381 in the form of a salt bridge as well as a hydrogen bond to nACE‐Tyr369. Despite this, SG18 is the weakest P2 carboxylic acid‐containing diprolyl‐derived inhibitor for nACE within this series of compounds. The highly nACE‐selective inhibitor 33RE also does not target nACE‐Arg381 [28]. This further supports the premise that the nACE‐Arg381 to cACE‐Glu403 substitution may not be an effective residue to target in the design of nACE‐selective inhibitors, due to effects away from the active site [37].

### Comparison with cACE‐specific inhibitor RXPA380

**Fig. 12:**
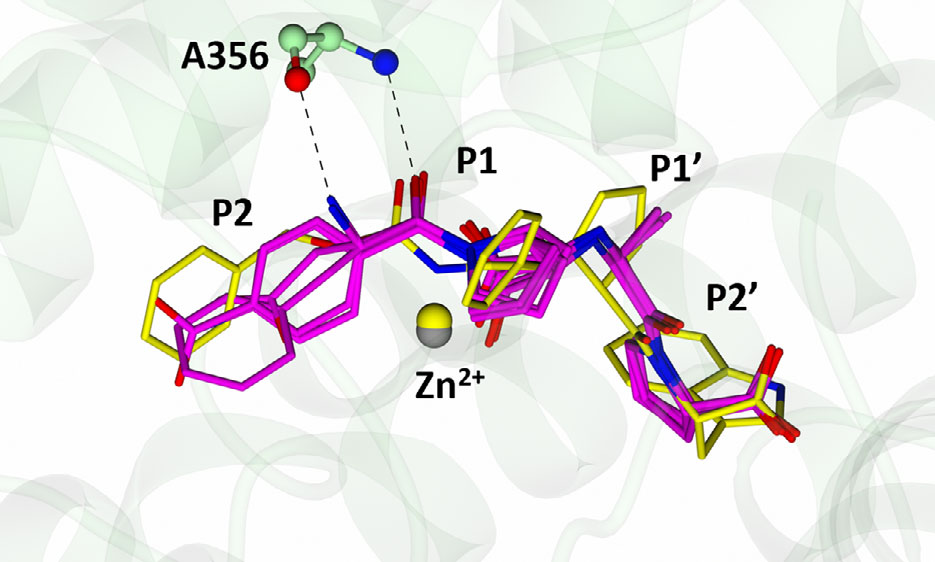
Comparison of cACE in complex with SG3, S15, SG18 and RXPA380. Superimposition of cACE:SG3, cACE:SG15, cACE:SG18 and cACE:RXPA380 (PDB code 2OC2) reveal similar positioning of the inhibitors within the active site, except for the P2 moiety and larger P2′ moiety in RXPA380. SG compounds are shown in magenta and RXPA380 in yellow. The zinc atoms from the cACE structures in complex with SG compounds are shown in grey and RXPA380 in yellow. Only cACE‐Ala356 of cACE:SG15 is shown for simplicity. Hydrogen bonds are shown by the dotted lines. Figure produced with CCP4MG.

RXPA380 is highly selective for cACE, with a nACE selectivity factor of 3 × 10^−4^ (Table 1). Two out of four of the compounds presented here have comparable dissociation constants for cACE as RXPA380 (SG3 and SG18) but lack the high domain‐selectivity of the former. To understand their lack of selectivity, we compared the cACE structures presented here to that of cACE:RXPA380. In each structure, hydrophobic contacts and hydrogen bonding, interactions between each inhibitor and cACE are conserved for the S2′, S1′ and S1, except RXPA380, which contains larger hydrophobic moieties capable of forming better contacts (Fig. 12). Given that SG3 and SG15 have higher affinity than RXPA380 (Table 1), the introduction of bulkier hydrophobic residues at the S2′, S1′ and S1 subsites does not significantly contribute to binding strength. However, it is important to consider the impact of such groups in nACE binding, in particular at the S2′ subsites. With respect to the S2 subsite, SG15 is most structurally similar to RXPA380, but displays a 55.93‐fold reduction in binding strength. The P2 ring of RXPA380 appears to be rotated ~90° compared to SG15, and the presence of the amine group in SG15 restricts the orientation of the P2 phenylalanine within the S2 subsite. With respect to SG3 and SG18, the P2 rings occupy the same orientation as the ring of RXPA380 but lack the length to position deeper into the S2 subsite to form hydrophobic interactions with cACE‐His410 and cACE‐Phe391 that are observed in RXPA380. Despite this, SG3 and SG18 bind cACE 762.72‐fold and 73.28‐fold stronger than SG15. Therefore, it appears that the inhibitors with a P2 ring moiety orientated similarly to RXPA380 (semi‐perpendicular to cACE‐Phe391) have lower dissociation constants, and the design of cACE inhibitors may benefit from hydrophobic moieties within the S2 subsite that can position perpendicular to cACE‐Phe391.

### The P2 amine of SG15, SG16, and SG17 may contribute to selectivity

The nACE‐selective compounds SG15, SG16 and SG17 contain an amide group within the P2 moieties that forms a hydrogen bond with nACE‐Ala334 (cACE‐Ala356) via the backbone nitrogen and oxygen atoms in nACE and cACE (Figs 11 and 12). In contrast, the cACE‐selective compounds, SG3 and SG18, lack the amine group and therefore only form a hydrogen bond with the nitrogen atom of nACE‐Ala334 (cACE‐Ala356). This suggests that the presence of the P2 amine may contribute to a difference in selectivity. Given that the hydrogen bonding to nACE‐Ala334 (cACE‐Ala356) appears to be conserved between nACE and cACE in complex with SG15, SG16 and SG17, the additional hydrogen bond to the oxygen atom of nACE‐Ala334 (cACE‐Ala356) is unlikely to contribute to the selectivity observed. Therefore, the polar/charged nature of the amine group within the S2 subsite may account for the differences in selectivity. The change of cACE‐Phe391 to nACE‐Tyr369 would explain the increased affinity of nACE for SG15, SG16 and SG17, within the S2 subsite, as the hydroxyl group provides greater stability for the polar/charged amine group. This suggests that introduction of an amine into the P2 moiety of ACE inhibitors favours nACE selectivity. However, we previously showed that SG2 (which contains only an amine in the P2 moiety) is cACE selective [30]. The substitution of cACE‐Glu403 to nACE‐Arg381 may explain the cACE selectivity of SG2, where the protonated amine group is stabilised by an electrostatic interaction with cACE‐Glu403. The above points indicate that the amine group does contribute to selectivity, but the effect of the group changes depending on the presence of additional functional groups that make up the P2 moieties. For example, the presence of the phenyl ring in SG15, and carboxylate groups of SG16 and SG17, may shield the charge of nACE‐Arg381 or cACE‐Glu403 from the charged amine group, and thus, the selectivity is more influenced by the nACE‐Tyr369 to cACE‐Phe391 substitution. Interestingly, the potent nACE‐selective compounds RXP407 and 33RE also contain an amine within their P2 moieties, which binds similarly to nACE‐Ala334 supporting the premise that an amine within the P2 moiety may influence selectivity due to the variable electrostatic potential of the S2 subsite between nACE and cACE.

## Conclusion

The crystal structures of nACE and cACE in complex with a series of diprolyl‐derived inhibitors (SG3, SG15, SG16, SG17 and SG18) were analysed in order to gain insights into domain‐specific ACE inhibition. The P1, P1′ and P2′ moieties are conserved across the series, with variable P2 moieties designed to target the nACE‐specific S2 subsite residues, nACE‐Arg381 and nACE‐Tyr369, with the exception of SG15, by hydrogen bonding (SG3) and/or a salt bridge (SG16, SG17 and SG18). Overall, for each inhibitor, nACE possesses a higher number of protein:ligand interactions in comparison to cACE via a number of hydrogen bonds, hydrophobic contacts and water‐mediated interactions. Interactions at the S1, S1′ and S2′ are conserved across the series, except for an additional water‐mediated interaction in nACE structures. Only SG16 was shown to be potently nACE selective (in a previous study), with the remaining inhibitors possessing nanomolar affinity but weak domain selectivity. Interestingly, SG3 and SG18 inhibitors, which were predicted to be better binders for nACE due to selective targeting of nACE‐Arg381 and nACE‐Tyr369, bound cACE more strongly despite possessing fewer and weaker direct protein:ligand interactions. nACE‐Arg381 was also shown to be highly flexible, likely only tightly binding SG18 due to its increased length enhancing its ability to form a salt bridge via its P2 carboxylic acid group. Despite this, of the compounds containing a P2 carboxylic acid group, SG18 is the weakest nACE inhibitor. This indicates that the targeting of nACE‐Arg381 for the design of nACE‐selective inhibitors is likely a poor strategy and may negatively influence selectivity due to the involvement of nACE‐Arg381 and nACE‐Arg90 in nACE cleft closure. These findings further illustrate the need to consider the impact of residues distal from the active site on inhibitor binding for the design of selective ACE inhibitors, particularly those involved in subdomain dynamics and cleft closure.

## Materials and methods

SG3, SG15, SG16, SG17 and SG18 were synthesised, purified and characterised as previously described [30].

### Protein expression and purification

Minimally glycosylated and truncated nACE (N389) and cACE (g1,3) (Ser‐1 to Pro‐633) human ACE were expressed in cultured mammalian CHO cells and purified as previously described [39, 40].

### Crystallisation and X‐ray crystallography

Both nACE and cACE were concentrated to 5 and 10 mg·mL^−1^, respectively, then mixed with the SG compounds in a 3.75 : 1 volume ratio of protein:SG compound (SG compounds at [4 mm] each). The complexes were left to equilibrate at room temperature (nACE) and on ice (cACE) for ~1 h prior to setting up crystallisation. Crystallisation was performed by hanging drop using 1 : 1 μL ratio of protein to 30% PEG 550 MME/PEG 20000, 0.1 m Tris/Bicine pH 8.5 and 60 mm divalent cations [Molecular Dimensions (Rotherham, UK) Morpheus A9] for nACE, and 0.1 m MIB pH 4.0, 5% glycerol, and 15% PEG 3350 for cACE. Crystals were mounted onto a cryoloop and flash cooled in liquid nitrogen for X‐ray diffraction data collection at 100 K. A total of 3600–7200 images were taken at 0.1° of oscillation with an exposure time of 0.004–0.04 s per image (depending on datasets). Raw images were indexed and integrated using DIALS [41], with subsequent data processing performed using the CCP4 suite [42], including data reduction with AIMLESS, phase estimation with Phaser [43] (using 6F9V and 6F9T [44] as the search model for nACE and cACE structures, respectively) and refinement with REFMAC5 [45] and Coot [46]. The inhibitors (SG3, SG15, SG16, SG17 and SG18), zinc ions, chloride ions and purification/crystallisation buffer reagents were added based on the Fo‐Fc Fourier difference map. The structures were validated using Molprobity [47] and figures created with CCP4MG [48].

## Conflict of interest

The authors declare no competing financial interests.

## Author contributions

KSG wrote the manuscript, performed all crystallographic analysis, analysed the data and edited the manuscript. GEC performed crystallisation, collected X‐ray diffraction data, analysed the data and edited the manuscript. SF and KC were involved in the design and production of the diprolyl compounds and edited the manuscript. EDS helped conceptualise the study, design the diprolyl compounds and edited the manuscript. KRA supervised the study, analysed the data and edited the manuscript.

### Peer review

The peer review history for this article is available at https://www.webofscience.com/api/gateway/wos/peer‐review/10.1111/febs.17384.

## Data Availability

The atomic coordinates and structure factors of nACE:SG3, nACE:SG15, nACE:SG17, nACE:SG18, cACE:SG3, cACE:SG15, cACE:SG16 and cACE:SG18 complexes have been deposited under accession codes 9GBP, 9GBQ, 9GBR, 9GBS, 9GBM, 9GBN, 9GBO and 9GBL, respectively, in the RCSB Protein Data Bank, www.pdb.org.
